# Supplementary material for: The PNUTS-PP1 complex acts as an intrinsic barrier to herpesvirus KSHV gene expression and replication
Source: Nat Commun. 2022 Dec 2;13:7447. doi: 10.1038/s41467-022-35268-4 (PMC9718767; doi:10.1038/s41467-022-35268-4)

**Supplementary information for: The PNUTS-PP1 complex acts as an intrinsic barrier to herpesvirus KSHV gene expression and replication**

Anne M. Devlin^1^, Ashutosh Shukla^1^, Julio C. Ruiz^1^, Spencer D. Barnes^2^, Ashwin Govindan^1^, Olga V. Hunter^1^, Anna M. Scarborough^1^, Iván D’Orso^1^, Nicholas K. Conrad^1^*

1 Department of Microbiology, UT Southwestern Medical Center, Dallas, TX, USA

2 Lyda Hill Department of Bioinformatics, UT Southwestern Medical Center, Dallas, TX, USA

*Correspondence: Nicholas.Conrad@UTSouthwestern.edu

**Supplementary Figures**


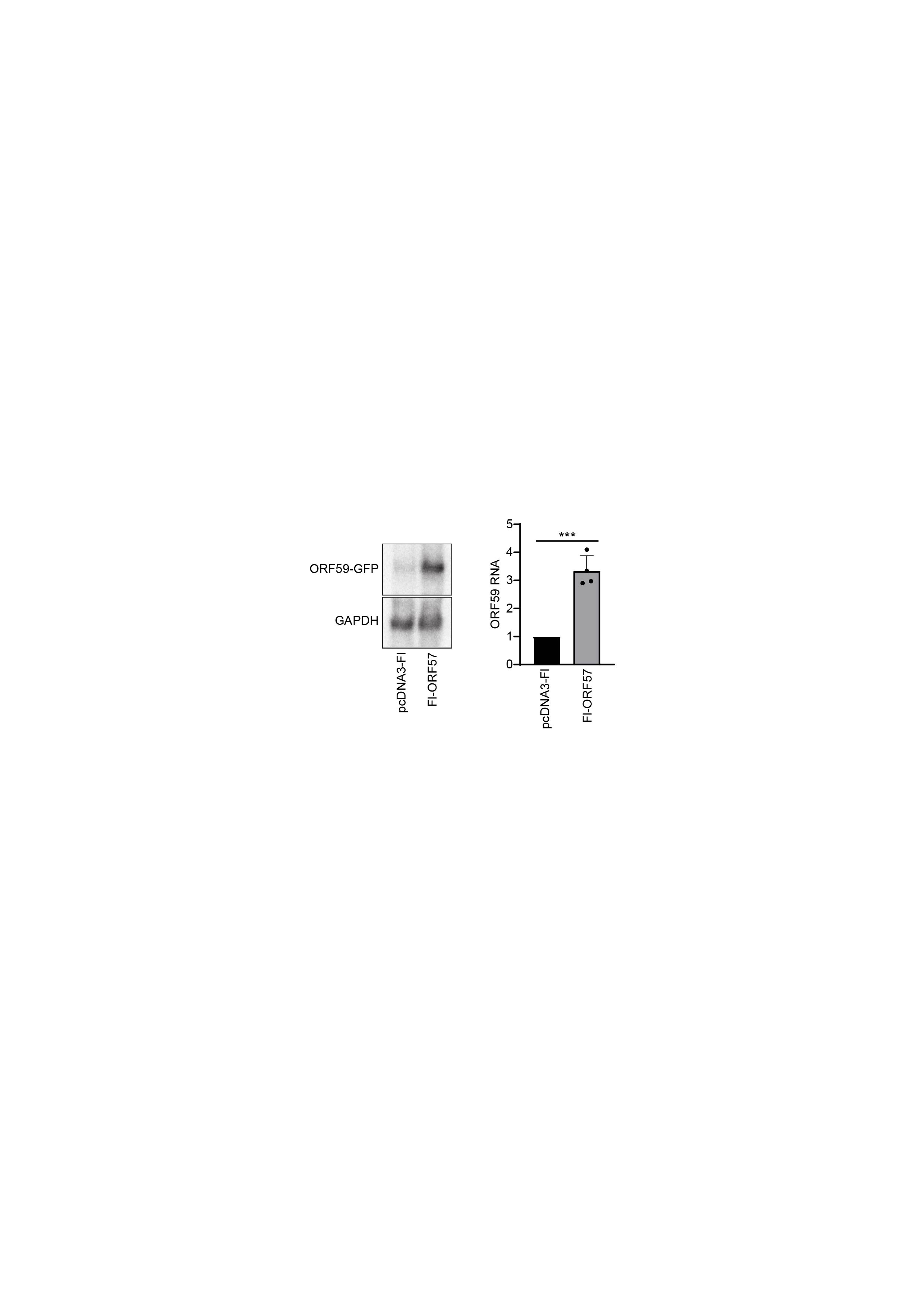


**Supplementary Figure 1 | Validation of integrated ORF59-GFP reporter in HCT116 cells.**

Northern blot with GFP probe and quantification of HCT116 ORF59-GFP-Hyg reporter line transfected with pcDNA3-Fl or Fl-ORF57. ORF59-GFP signal was normalized to GAPDH and quantified relative to pcDNA3-Fl. Error bars are mean with standard deviation, asterisks denote Student’s two-tailed unpaired t-test (****p*=0.0001), (*n*=4). Source data are provided as a Source Data file.


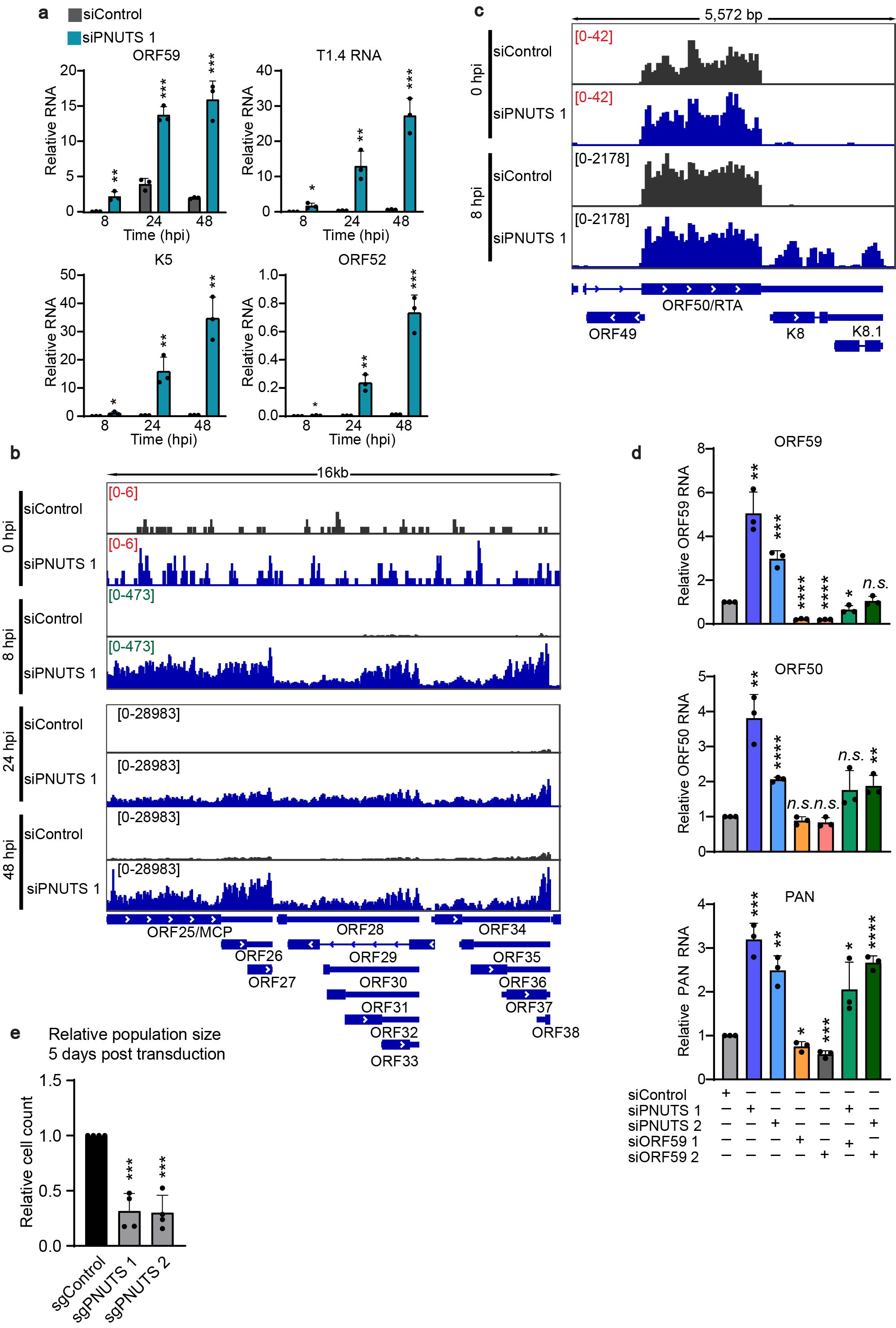


**Supplementary Figure 2 | Viral gene expression in iSLK and TREx-shRNA-BCBL1 cells.**

**a,** RT-qPCR (as in Figure 2b) of siControl or siPNUTS 1 treated iSLK cells harvested at 8, 24, or 48 hours after lytic induction in iSLK cells. Values were normalized to beta-actin mRNA. Error bars are mean with standard deviation, asterisks denote Student’s two-tailed unpaired t-test between siControl and siPNUTS 1 at each time point (ORF59 8hpi ***p*=0.0054, 24hpi ****p*=0.0003, 48hpi ****p*=0.0008; T1.4 RNA 8hpi **p*=0.0168, 24hpi ***p*=0.0067, 48hpi ****p*=0.0007; K5 8hpi **p*=0.0489, 24hpi ***p*=0.0067, 48hpi ***p*=0.0015; ORF52 8hpi **p*=0.0417, 24hpi ***p*=0.0024, 48hpi ****p*=0.0006), (*n*=3). **b,** IGV browser view of all timepoint samples taken at a representative region of the KSHV genome, 0hpi and 8hpi scales distinct from 24hpi and 48hpi. Only transcripts from the “top” strand (left to right) are included. **c,** IGV browser view of ORF50 from one biological replicate. RNAs from both the integrated doxycycline-inducible ORF50 transgene and RNA from the bacmid-encoded ORF50 map to the displayed region, so we cannot distinguish whether expression is from exogenous or viral genome. Note that the scales for 0hpi and 8hpi differ. **d,** RT-qPCR of ORF59, ORF50, and PAN RNA levels at 24hpi in iSLK cells after knockdown of PNUTS, ORF59, or PNUTS and ORF59 together. Values normalized to beta-actin. Error bars are mean with standard deviation, asterisks denote Student’s two-tailed unpaired t-test against siControl sample. ORF59 graph *p* values: (siPNUTS 1 ***p*=0.0020, siPNUTS 2 ****p*=0.0007, siORF59 1 *****p*=1.1x10^-6^, siORF59 2 *****p*=1.5x10^-7^, siPNUTS 1/ siORF59 1 **p*=0.0305, siPNUTS 2/ siORF59 2 *n.s.* *p*=0.6828). ORF50 graph *p* values: (siPNUTS 1 ***p*=0.0020, siPNUTS 2 *****p*=9.9x10^-6^, siORF59 1 *n.s. p*=0.1471, siORF59 2 *n.s. p*=0.1073, siPNUTS 1/ siORF59 1 *n.s. p*=0.0757, siPNUTS 2/ siORF59 2 ***p*=0.0075). PAN graph *p* values: (siPNUTS 1 ****p*=0.0005, siPNUTS 2 ***p*=0.0015, siORF59 1 **p*=0.0176, siORF59 2 ****p*=0.0008, siPNUTS 1/ siORF59 1 **p*=0.0426, siPNUTS 2/ siORF59 2 *****p*=5.2x10^-5^), (*n*=3). **e,** Relative live cell counts by trypan blue staining of TREx-RTA-BCBL1 cells after transduction with non-targeting vs two independent anti-PNUTS sgRNAs at five days post-transduction. Error bars are mean with standard deviation, asterisks denote Student’s two-tailed unpaired t-test (siControl vs siPNUTS 1 ****p*=0.0001, siControl vs siPNUTS 2 ****p*=0.0001), (*n*=4). Source data are provided as a Source Data file.


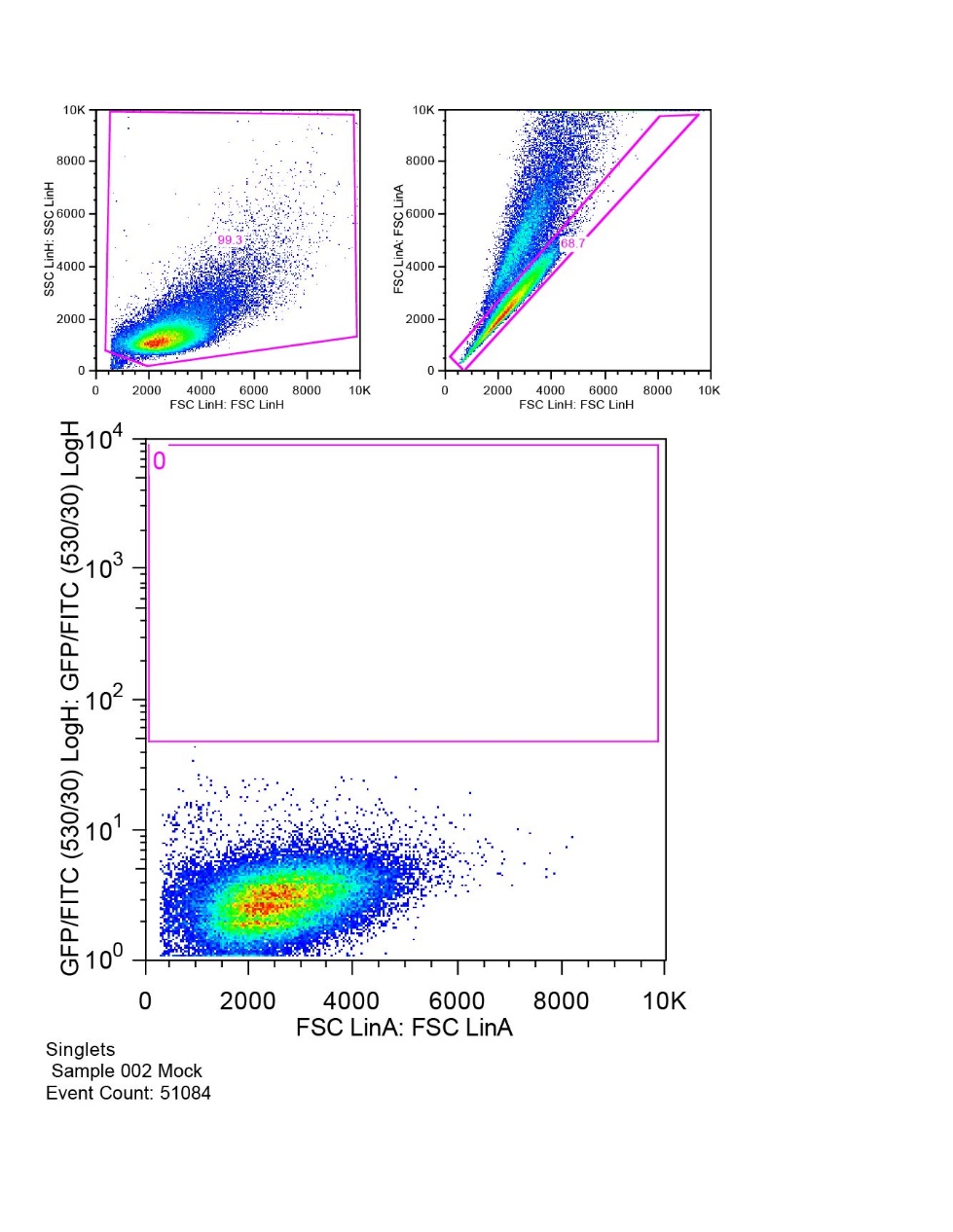


**Supplementary Figure 3 | Gating strategy for spinfection flow cytometry assays.**

Debris was excluded by primary gating with FSC-H/SSC-H (upper left panel), then singlets were selected by FSC-H/FSC-A plots (upper right panel). Percent GFP positive was plotted by GFP/FITC-H/FSC-A (lower panel). Gates are as shown in pink boxes on figure.


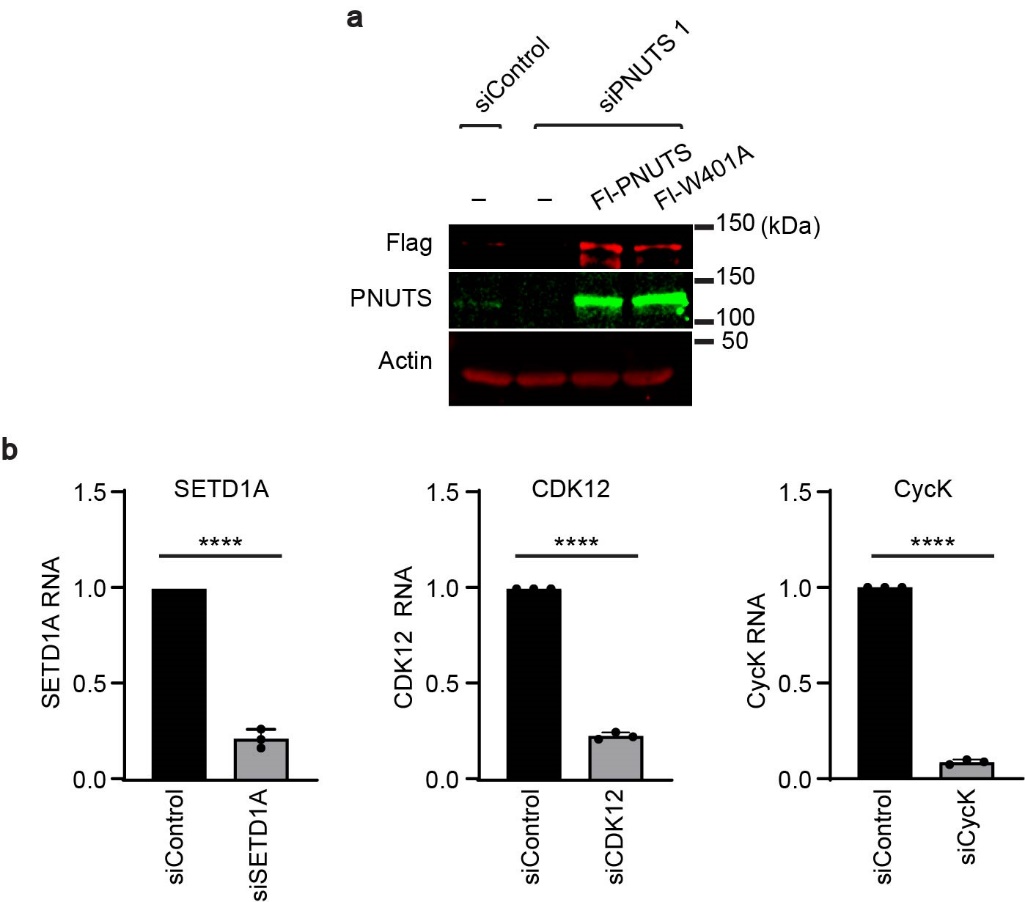


**Supplementary Figure 4 | siRNA knockdowns and rescue validations.**

**a,** Representative western blot of PNUTS siRNA depletion and rescue with siRNA-refractory Fl-PNUTS or Fl-W401A constructs. Actin serves as a loading control. **b** RT-qPCR of knockdowns for SETD1A, CDK12, and CycK mRNA levels in their respective knockdown samples, normalized to beta-actin and displayed relative to siControl samples. Error bars are mean with standard deviation, asterisks denote Student’s two-tailed unpaired t-test (siControl vs siSETD1A *****p*=1.0x10^-5^, siControl vs siCDK12 *****p*=2.1x10^-7^, siControl vs siCycK *****p*=2.8x10^-8^), (*n*=3). Source data are provided as a Source Data file.


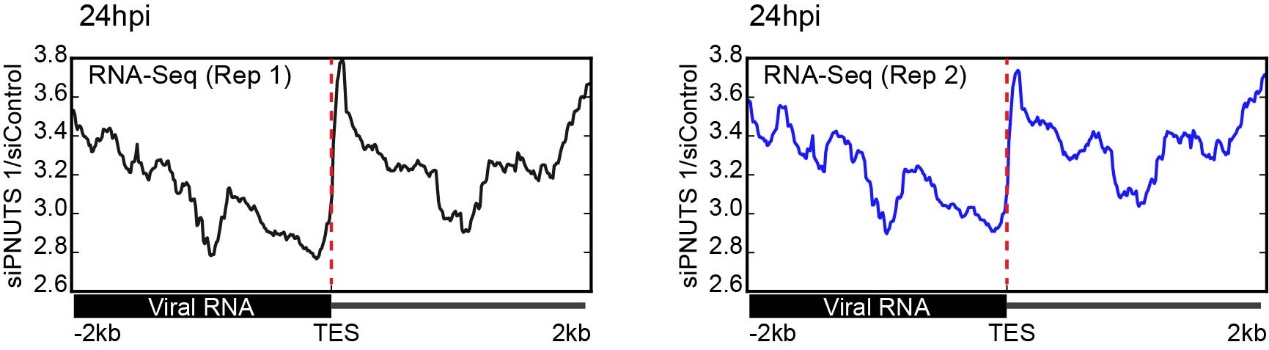


**Supplementary Figure 5 | Metagenes from independent RNA-Seq replicates.**

Metagene plots of RNA-seq data aligned to the viral TESs of two independent biological replicates (24hpi; third replicate in Fig. 5e).


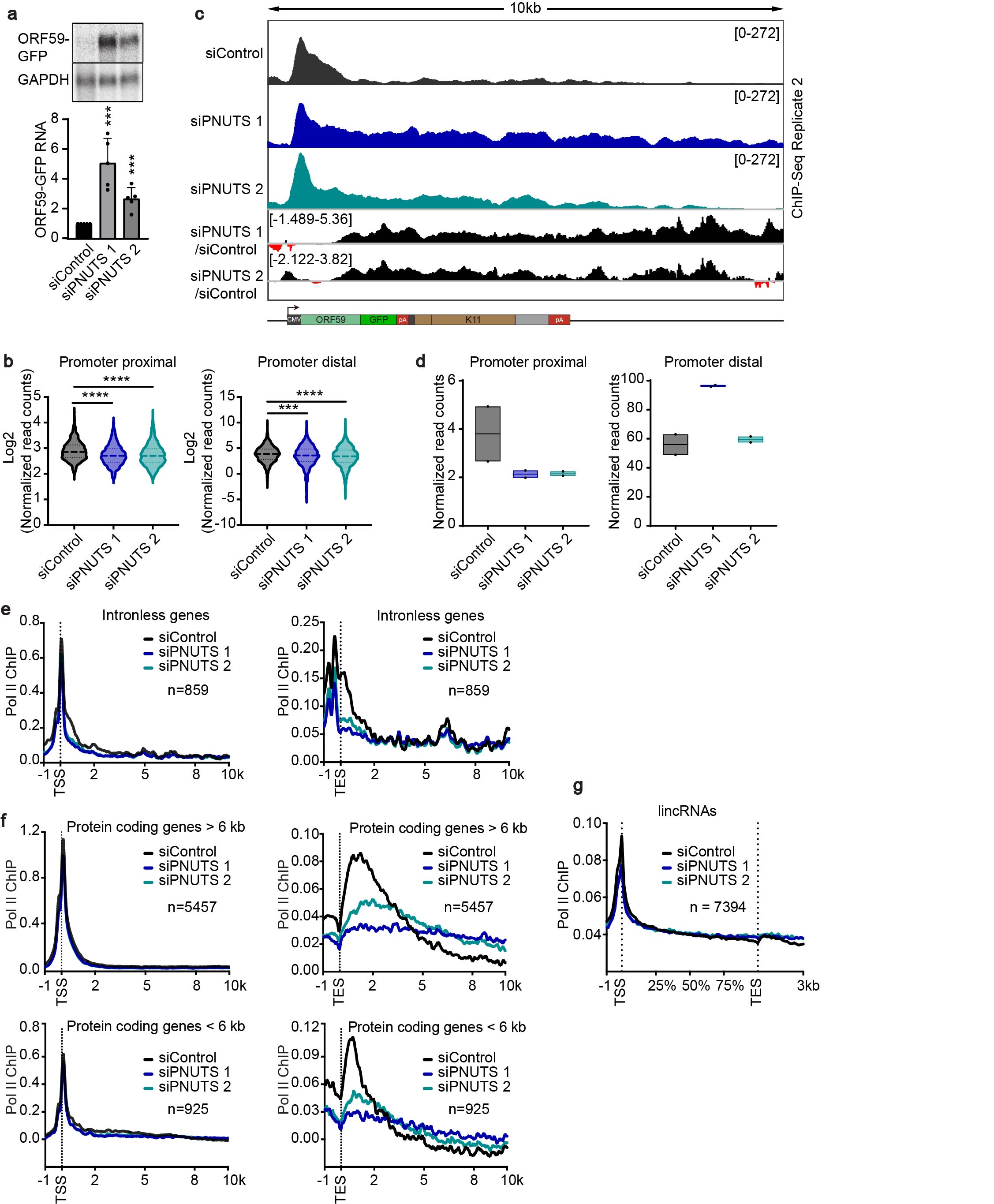


**Supplementary Figure 6 |** **New reporter validation and ChIP-Seq.**

**a,** Northern blot and quantification of ORF59-GFP RNA levels in a clonal line with integrated ORF59-GFP-K11, with GFP probe. Values were normalized to GAPDH and are displayed relative to siControl sample. Error bars are mean with standard deviation, asterisks denote Student’s two-tailed unpaired t-test (siControl vs siPNUTS 1 ****p*=0.0006, siControl vs siPNUTS 2 ****p*=0.0010), (*n*=5). **b,** Input normalized read counts (Log2scale) for promoter proximal pol II and promoter distal pol II of protein-coding endogenous genes (n=1358). The thick dashed line indicates median, and two thin dashed lines below and above show 1^st^ and 3^rd^ quartile respectively. Asterisks denote two-tailed Wilcoxon rank sum tests for pairwise comparisons between samples. (Promoter proximal siControl vs siPNUTS 1 or vs siPNUTS 2 *****p*<0.0001; Promoter distal siControl vs siPNUTS 1 ****p*=0.0004, siControl vs siPNUTS 2 *****p*<0.0001). **c,** IGV browser view of the integrated ORF59-GFP-K11 reporter from an independent biological replicate of pol II ChIP-seq from control or PNUTS-depleted cells. Bottom tracks are relative (log2 ratio) pol II occupancy comparing PNUTS-depleted samples to non-targeting control; black and red denote higher and lower signal in PNUTS-depleted samples, respectively. **d,** Input normalized read counts for promoter proximal pol II and promoter distal pol II on integrated reporter. Black line inside the boxplot indicates the mean values. The black dots show data points from two replicates. **e,** Metagenes of pol II profiles around TSS or TES on human intronless genes from one biological replicate. **f,** Metagene plots around TSS and TES for protein coding genes greater than 6kb in length vs. genes between 1 and 6kb, from one biological replicate. **g,** Metagene plot from TSS to TES of lincRNAs, from one biological replicate. Source data are provided as a Source Data file.


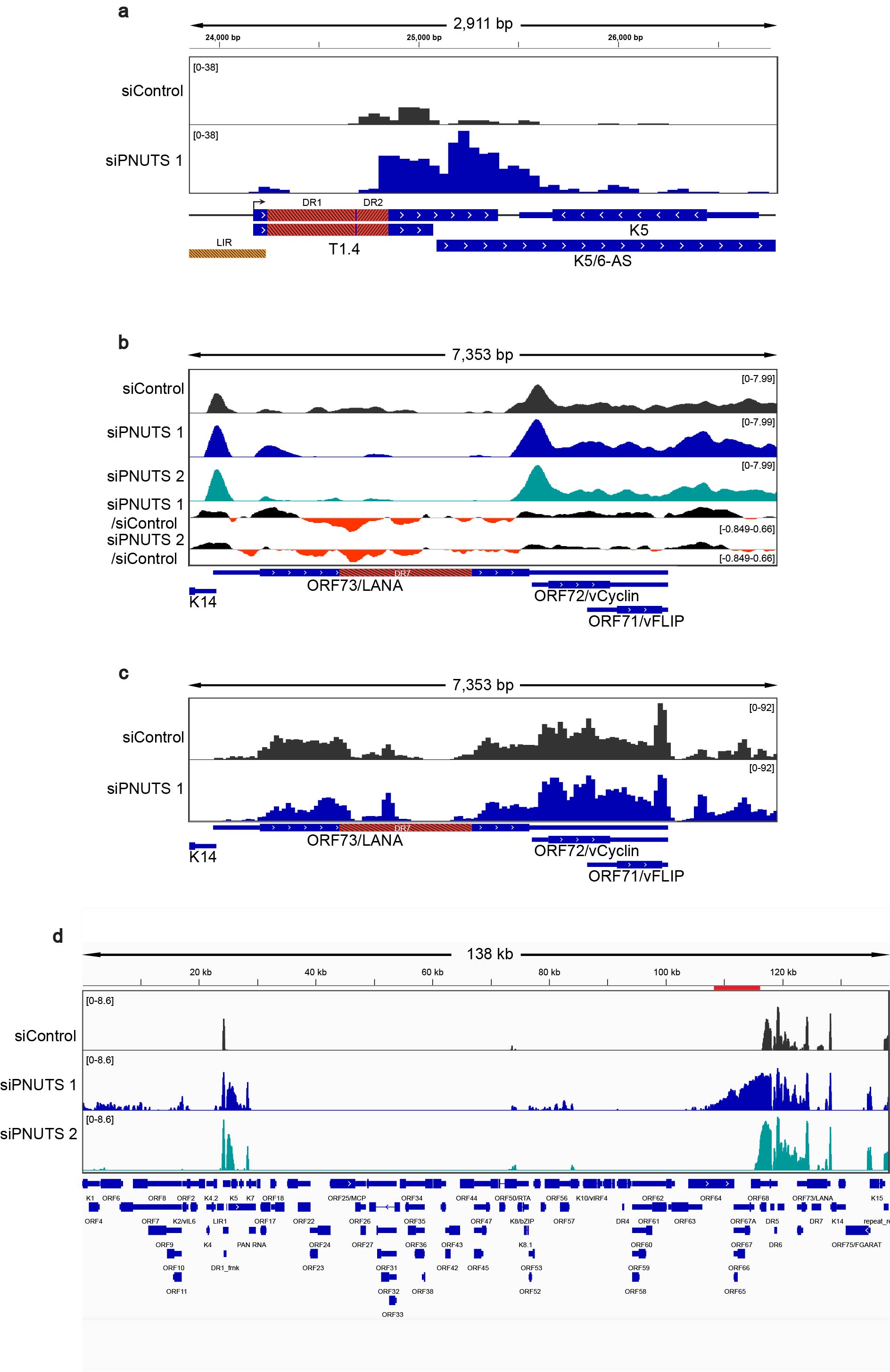


**Supplementary Figure 7 | RNA and ChIP-Seq profiles in uninduced iSLK cells**

**a,** IGV browser view of RNA-Seq in uninduced iSLK cells of the T1.4 locus. **b,** IGV browser view of the latency genes ORF73, ORF72, and ORF71 from one pol II ChIP-Seq replicate in uninduced iSLK cells. **c,** RNA-Seq as in **a,** view of the latency locus in uninduced iSLK cells. **d,** IGV browser view of the entire KSHV genome from one replicate of total pol II ChIP-Seq in uninduced iSLK cells. The red bar marks a region of pol II readthrough from the latent kaposin locus in the siPNUTS-treated cells.

**Supplementary Tables**

**Supplementary Table 1 | Oligonucleotides**

Sequences or commercial identifiers for all primers, oligonucleotide probes and siRNAs used in this study.

| **qPCR Primers** |  |  |  |  |
| --- | --- | --- | --- | --- |
| Amplicon | FWD Name | FWD Sequence | REV Name | REV Sequence |
| ORF59 (A) [4SU-qPCR assays] | NC572 | AGGCAGCCGGAATTGGA | NC573 | AAGGGACCAACTGGTGTGAGA |
| K11 (B) | NC4201 | GGCATCACGGGAAATTGG | NC4202 | CTGTTTCCTGGCGGC |
| Unspliced Actin for 4SU assays | NC1230 | CTTCAACACCCCAGCCATGT | NC1231 | CCAGAGGCGTACAGGGATAGC |
| ORF59 [all other ORF59 qPCRs] | NC1471 | AGGAATACGTCCGTCGGTAG | NC1472 | TATGCCAGCGTCGAGTACAT |
| PAN | NC702 | GCTCGCTGCTTGCCTTCTT | NC703 | CCAAAAGCGACGCAATCAA |
| LANA/ORF73 | NC1721 | CTCCGGAAAGATGTGACCTT | NC1722 | GGGACTTCCAGGTATAGGCA |
| ORF58 | NC3030 | GTAAGGTCACAAACACGACAAC | NC3031 | CAAAGGAGGAGGGCCATATT |
| K5 | NC1361 | TAAGCACTTGGCTAACAGTGT | NC1362 | GGCCACAGGTTAAGGCGACT |
| T1.4 | NC3716 | ATTCAAAGGGGGCACAGAGG | NC3717 | CCTGAAACGTCGCCCAAATC |
| ORF52 | NC3564 | GACCTTACGATGGAAGACCTAAC | NC565 | CAGTGGATCCCAGTGCTTT |
| ORF50 | NC712 | ACCGGTCTACCTTCCGAGGAT | NC713 | GGCGAGCTGACCCTTGGT |
| Actin mRNA | NC1224 | ACCCAGCACAATGAAGATCA | NC1225 | CTCGTCATACTCCTGCTTGC |
| 7SK | NC1164 | TAAGAGC TCGGATGTGAGGGCGATCTG | NC1165 | CGAATTCGGAGCGGTGAGGGAGGAAG |
| GAPDH | NC1234 | CTCTCTGCTCCTCCTGTTCGA | NC1235 | CACCTGGCGACGCAAAA |
| SETD1A | NC3239 | CGGAAGAAGAAGCTCCGATTT | NC3240 | GGATGTTCTGACCCACGTATTC |
| CDK12 | NC3241 | GGGATTGTGTGTGGTTTCTTTC | NC3242 | GGCCACTCTGTTTCCCTTATAG |
| CycK | NC3243 | GATACCCTGGCAACTGGAATAA | NC3244 | CTTCTACTTTCCCAGCCAGAAA |
|  |  |  |  |  |
| **Primers to synthesize northern blot probes** | |  |  |  |
| Probe | FWD Name | FWD Sequence | REV Name | REV Sequence |
| ORF59 | NC570 | CGTGACCCTCCTGTCTAAAATCA | NC474 | CGCGTAATACGACTCACTATAGGGTCAAATCAGGGGGTTAAATG |
| GAPDH | NC1549 | CCTGCCGTCTAGAAAAACCTG | NC1550 | CGCGTAATACGACTCACTATAGGGGGTTGAGCACAGGGTACTTTA |
| GFP | NC2909 | ACCACATGAAGCAGCACGAC | NC4001 | TAATACGACTCACTATAGGGATCTTGAAGTTCACCTTGATG |
| HygromycinR | NC3657 | ATGAAAAAGCCTGAACTCACCG | NC3658 | CGCGTAATACGACTCACTATAGGGTCAAGACCAATGCGGAGCATAT |
|  |  |  |  |  |
| **Cloning primers** |  |  |  |  |
| Construct | FWD Name | FWD Sequence | REV Name | REV Sequence |
| pAAVS1-ORF59-GFP | NC2424 | AAACGTTTCGAAGCCGCCACCATGCCTGTGGATTTTCACTAT | NC2422 | GCCCAAGGTACCAATCAGGGGGTTAAATGTGGT |
| pAAVS1-ORF59-GFP-K11 | NC3761 | ACATCCACCGGTTTGAATAATACATGTGTTTTTCTTGGTTTG | NC3762 | GGCCGCAGCGATCGCGGCACAAGCTTGTCACCCAC |
| pLentiCRISPRv2TC Part A | NC3180 | GTTTTAGAGCTAGAAATAGCAAG | NC3181 | CACTCCTTTCAAGACGAAGCTAGCGAATTCAAAAAAG |
| pLentiCRISPRv2TC Part B | NC3182 | AGCTTCGTCTTGAAAGGAGTGGGAATT | NC3183 | GATGCTGTACTTCTTGTCCAT |
| Fl-PNUTS Part A | NC3380 | GGAGGGATCCATGGGTTCGGGTCCCATAG | NC3632 | TATCTAACTAACGTTCTTGGCTGTTACTTCCAGGGGCAAGGTTCTTGTCTTTTCCTTCCAG |
| Fl-PNUTS Part B | NC3631 | GTAACAGCCAAGAACGTTATATCCAGGCTGAGCGGGAGA | NC3381 | GGAG CTCGAG CTA GGGCAGGGGGGGCCCATT |
| FL-W401A Part A | NC3380 | GGAGGGATCCATGGGTTCGGGTCCCATAG | NC3655 | GCCTTCCTCAGGAGCTGTCACACTTTTCCT |
| FL-W401A Part B | NC3654 | GTGACAGCTCCTGAGGAAGGCAAACTGAG | NC3381 | GGAGCTCGAGCTAGGGCAGGGGGGGCCCATT |
|  |  |  |  |  |
| **siRNAs** | Source | Catalogue number | ID number |  |
| Negative Control No. 2 siRNA | Thermo Fisher | Cat#: 4390846 | N/A |  |
| ORF59 1 | Thermo Fisher | Custom design | siRNA ID#: 555960 | Sense sequence: AAAGGUUCCCGAUUCUAUAtt |
| ORF59 2 | Thermo Fisher | Custom design | siRNA ID#: 555962 | Sense sequence: GCAAGAUCAUUGAUUUCAAtt |
| PNUTS 1 | Thermo Fisher | Cat#: 4427037 | s328 |  |
| PNUTS 2 | Thermo Fisher | Cat#: 4427037 | s329 |  |
| SKIV2L2 (MTR4) 1 | Thermo Fisher | Cat#: 4427037 | s23983 |  |
| SKIV2L2 (MTR4) 2 | Thermo Fisher | Cat#: 4427037 | s23984 |  |
| ZFC3H1 1 | Thermo Fisher | Cat#: 4427037 | s47012 |  |
| ZFC3H1 2 | Thermo Fisher | Cat#: 4427037 | s47013 |  |
| ARS2 1 | Thermo Fisher | Cat#: 4427037 | s28375 |  |
| ARS2 2 | Thermo Fisher | Cat#: 4427037 | s28376 |  |
| SETD1A 1 | Thermo Fisher | Cat#: 4427037 | s18788 |  |
| SETD1A 2 | Thermo Fisher | Cat#: 4427037 | s18789 |  |
| CDK12 1 | Thermo Fisher | Cat#: 4427037 | s28621 |  |
| CDK12 2 | Thermo Fisher | Cat#: 4427037 | s28622 |  |
| CycK 1 | Thermo Fisher | Cat#: 4427037 | s16798 |  |
| CycK 2 | Thermo Fisher | Cat#: 4427037 | s16799 |  |
|  |  |  |  |  |
| **sgRNA / shRNA** | FWD Name | FWD Sequence | REV Name | REV Sequence |
| sgControl | NC3198 | CACCGTGTTCTACTTTCGAAGTTAA | NC3199 | AAACTTAACTTCGAAAGTAGAACAC |
| sgPNUTS 1 | NC3323 | CACCGCCAGCCGCCAGTCTTACCAG | NC3324 | AAACCTGGTAAGACTGGCGGCTGGC |
| sgPNUTS 2 | NC3325 | CACCGCCCACTTTAGACTCACGCTG | NC3326 | AAACCAGCGTGAGTCTAAAGTGGGC |
|  |  |  |  |  |
| shPNUTS | NC4006 | CCGGCACCAGAAATATTGGTCAAATCTCGAGATTTGACCAATATTTCTGGTGTTTTTG | NC4007 | AATTCAAAAACACCAGAAATATTGGTCAAATCTCGAGATTTGACCAATATTTCTGGTG |

**Supplementary Table 2 | Antibodies and plasmids**

Sources, catalog numbers and dilutions for all antibodies used in this study as well as all plasmids.

| **Antibodies** | **Source** | **Identifier** | **Dilution (Western blotting)** |
| --- | --- | --- | --- |
| Rabbit polyclonal anti-PNUTS | Bethyl Laboratories | Cat#: A300-439A-M | (1:1000) |
| Mouse monoclonal anti-PNUTS | BD Biosciences | Cat#: 611060 | (1:500) |
| Rabbit polyclonal anti-CPSF73 | Bethyl Laboratories | Cat#: A301-091A | (1:1000) |
| Mouse monoclonal anti-beta actin | Abcam | Cat#ab6276; RRID:AB_2223210 | (1:5000) |
| Goat anti-mouse | IRDye | Cat#:926-68020; RRID:AB_10706161 | (1:10,000) |
| Goat anti-mouse | IRDye | Cat#:926-32210; RRID:AB_621842 | (1:10,000) |
| Goat anti-rabbit | IRDye | Cat#: 926-32211; RRID:AB_621843 | (1:10,000) |
| Goat anti-rabbit | IRDye | Cat#: 926-68021; RRID:AB_10706309 | (1:10,000) |
| Normal Rabbit IgG polyclonal | Millipore | Cat#: 12-370 |  |
| Rabbit polyclonal anti-RPB3 | Millipore | Cat#: ABE999 |  |
| Rabbit polyclonal anti-Flag | Sigma | Cat#: F7425; RRID:AB_439687 | (1:1000) |
|  |  |  |  |
|  |  |  |  |
| **Plasmids** | **Name** | **Source** | **Identifiers** |
| Plasmid library: Human CRISPR Knockout Pooled Library (Brunello) |  | Drs. David Root and John Doench Doench et al., 2016 | RRID: Addgene#73179 |
| Plasmid: pcDNA3-Fl | pNC226 | Sahin et al., 2010 | N/A |
| Plasmid: pcDNA3 | pNC202 | Thermo Fisher | Cat#: V79020 |
| Plasmid: Fl-ORF57 | pNC561 | Sahin et al., 2010 | N/A |
| Plasmid: psp7SK for SP6-driven northern probe template | pNC491 | Dr. Joan Steitz |  |
|  |  |  |  |
| Plasmid: AAVS1 1L TALEN | pNC1050 | Dr. Feng Zhang Sajana et al., 2014 | RRID: Addgene #35431 |
| Plasmid: AAVS1 1R TALEN | pNC1051 | Dr. Feng Zhang Sajana et al., 2014 | RRID: Addgene #35432 |
| Plasmid: AAVS1-CMV-GFP-Hyg | pNC1049 | Dr. Joshua Mendell Manjunath et al., 2019 | N/A |
| Plasmid: AAVS1-ORF59-GFP-Hyg | pNC1115 | This paper |  |
| Plasmid: AAVS1-ORF59-GFP-K11 | pNC1461 | This paper |  |
|  |  |  |  |
| Plasmid: pMD2.G | pNC1091 | Dr. Didier Trono | RRID: Addgene #12259 |
| Plasmid: psPAX2 | pNC1092 | Dr. Didier Trono | RRID: Addgene #12260 |
| Plasmid: pLentiCRISPRv2 | pNC1090 | Dr. Feng Zhang | RRID: Addgene #52961 |
| Plasmid: pLentiCRISPRv2TC | pNC1213 | This paper |  |
| Plasmid: pLentiCRISPRv2TC sgRNA non-targeting | pNC1217 | This paper |  |
| Plasmid: pLentiCRISPRv2TC sgPNUTS #1 | pNC1274 | This paper |  |
| Plasmid: pLentiCRISPRv2TC sgPNUTS #2 | pNC1275 | This paper |  |
|  |  |  |  |
| Plasmid: pLKO-Tet-On-puro | pNC1548 | Dr. Dmitri Wiederschain | RRID: Addgene #21915 |
| Plasmid: pLKO-Tet-On-shRNA-Control | pNC1547 | Dr. Roland Friedel | RRID: Addgene #98398 ; http://n2t.net/addgene:98398 ; |
| Plasmid: pLKO-Tet-On-shRNA-PNUTS | pNC1552 | This paper | shRNA sequence: CACCAGAAATATTGGTCAAAT |
|  |  |  |  |
| Plasmid: pcDNA3.1+/C-(K)-DYK PNUTS ORF | pNC1399 | GenScript | ORF Clone ID: OHu03101D |
| Plasmid: Fl-PNUTS | pNC1412 | This paper |  |
| Plasmid: Fl-W401A | pNC1418 | This paper |  |

**Supplementary Table 3 | KSHV TSS/TES annotations**

Transcript start and end sites for KSHV open reading frames, + positive strand, - negative strand. Annotated sites from Arias *et al.* 2014[^32^](#_ENREF_32) and Majerciak *et al.* 2013[^33^](#_ENREF_33).

| **Chromosome** | **TSS** | **TES** | **Name** | **Strand** |
| --- | --- | --- | --- | --- |
| GQ994935.1 | 38 | 2956 | K1_ORF4 | + |
| GQ994935.1 | 3137 | 7013 | ORF6 | + |
| GQ994935.1 | 15610 | 17054 | ORF11_8_9_10 | + |
| GQ994935.1 | 17960 | 17163 | vIL6 | - |
| GQ994935.1 | 21099 | 18575 | K3 _K3A_ORF70 | - |
| GQ994935.1 | 21898 | 21274 | K4_4.1 | - |
| GQ994935.1 | 24175 | 25394 | 1.4 kb | + |
| GQ994935.1 | 26692 | 25501 | K5 | - |
| GQ994935.1 | 27472 | 26843 | K6 | - |
| GQ994935.1 | 28616 | 29690 | PAN_K7_K5/6AS | + |
| GQ994935.1 | 31536 | 30563 | ORF17.5_17 | - |
| GQ994935.1 | 29770 | 30566 | ORF16 | + |
| GQ994935.1 | 32242 | 33273 | ORF18 | + |
| GQ994935.1 | 40334 | 39048 | ORF23_24 | - |
| GQ994935.1 | 35120 | 39147 | ORF21_22 | + |
| GQ994935.1 | 42378 | 48596 | ORF25_26_27 | + |
| GQ994935.1 | 48748 | 53916 | ORF28_30_31_32_33 | + |
| GQ994935.1 | 54379 | 58691 | ORF34_35_36_37_38 | + |
| GQ994935.1 | 60050 | 58701 | ORF39 | - |
| GQ994935.1 | 63108 | 62232 | ORF42_43 | - |
| GQ994935.1 | 60124 | 62375 | ORF40/41 | + |
| GQ994935.1 | 64708 | 67134 | ORF44 | + |
| GQ994935.1 | 68683 | 67140 | ORF45_46_47_45.1 | - |
| GQ994935.1 | 72354 | 71432 | ORF49 | - |
| GQ994935.1 | 77063 | 76523 | ORF52_53 | - |
| GQ994935.1 | 71374 | 76554 | ORF50_K8_K8.1 | + |
| GQ994935.1 | 79525 | 78521 | ORF55 | - |
| GQ994935.1 | 77483 | 78588 | ORF54 | + |
| GQ994935.1 | 81819 | 83453 | ORF57 | + |
| GQ994935.1 | 79252 | 83490 | ORF56 | + |
| GQ994935.1 | 85026 | 83605 | K9 | - |
| GQ994935.1 | 88727 | 85823 | K10 | - |
| GQ994935.1 | 91211 | 89190 | K10.5 | - |
| GQ994935.1 | 93944 | 91573 | K11 | - |
| GQ994935.1 | 96613 | 94287 | ORF58 _59_60_61_62 | - |
| GQ994935.1 | 112340 | 111632 | ORF65_66_67_67.5 | - |
| GQ994935.1 | 114587 | 117240 | ORF68_69 | + |
| GQ994935.1 | 119205 | 117250 | K12 | - |
| GQ994935.1 | 124220 | 122513 | ORF71_72_73 | - |
| GQ994935.1 | 134923 | 130850 | ORF75 | - |
| GQ994935.1 | 128171 | 130870 | K14_ORF74 | + |

**Source data for supplementary information – uncropped gels / blots:**

**Supplementary data figure 1**


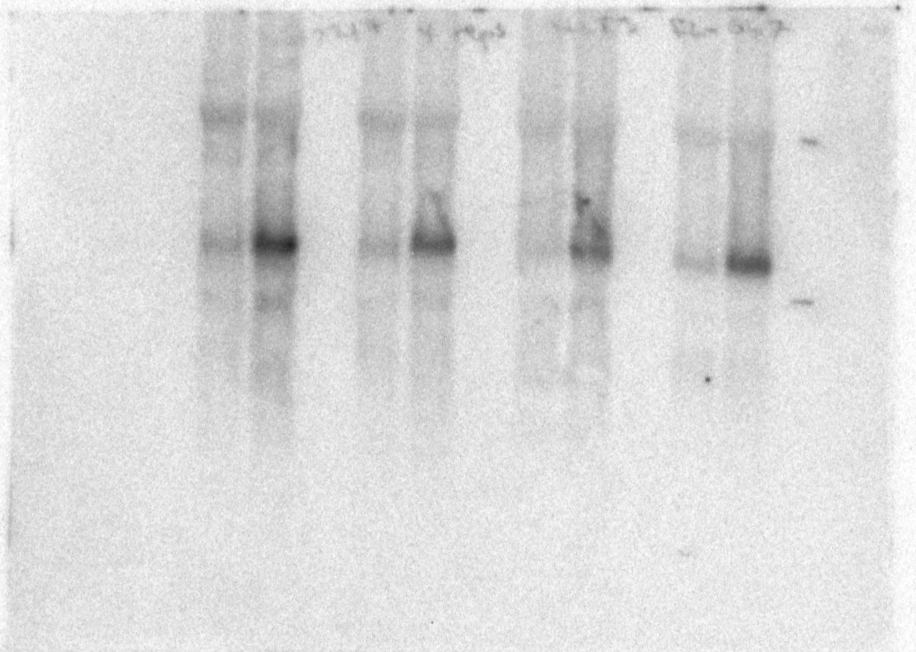
ORF59 probe (four replicates):

GAPDH probe:


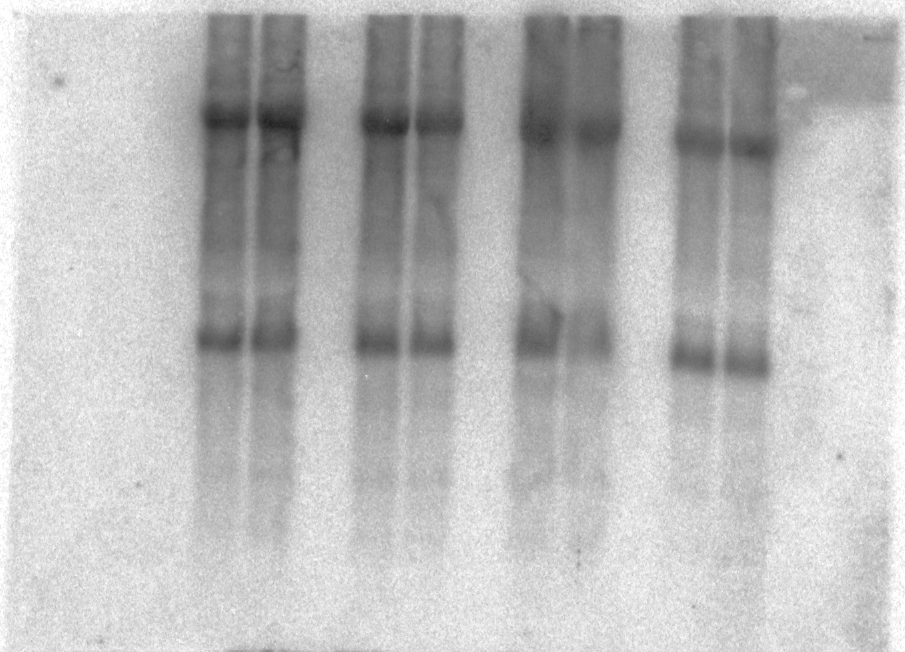


**Supplementary data figure 4a**

Rb anti Flag antibody:


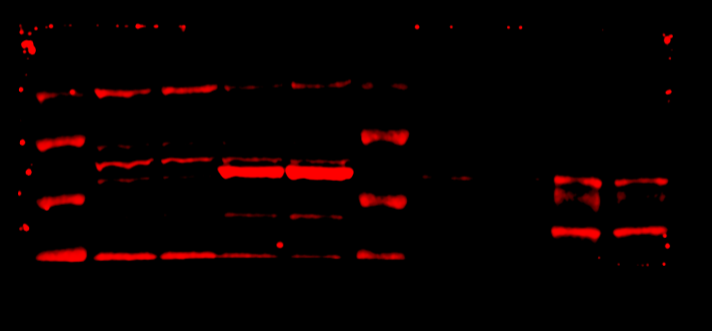


Lanes used in figure denoted with white box:


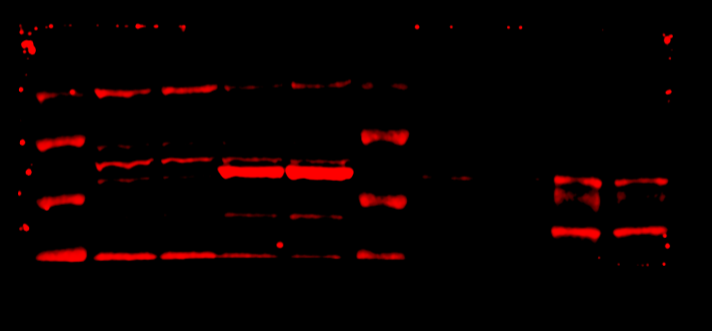


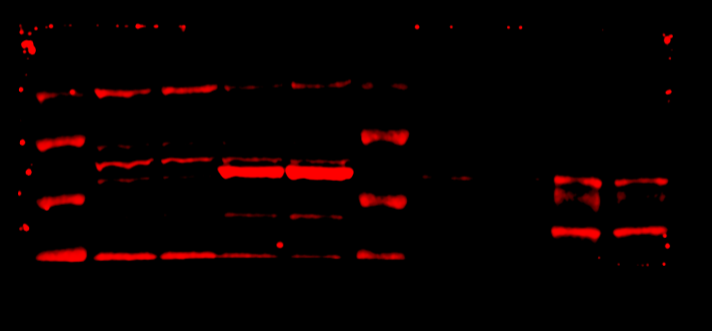


Ms anti PNUTS:


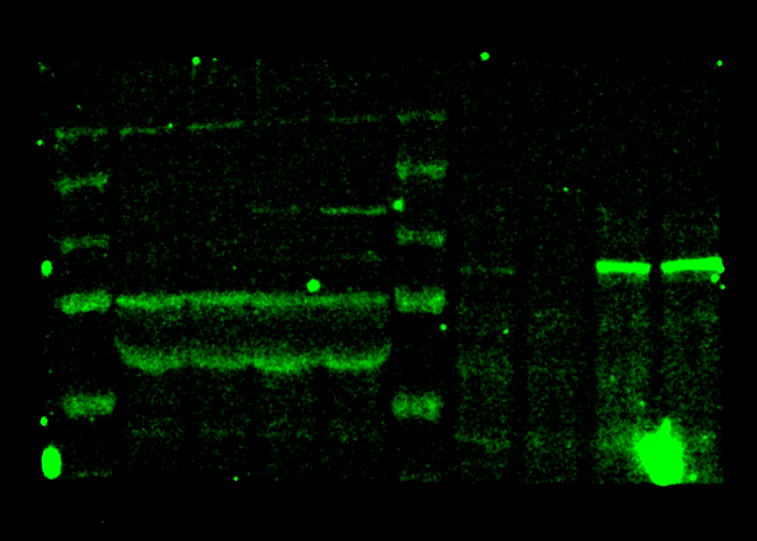


Ms anti Actin:


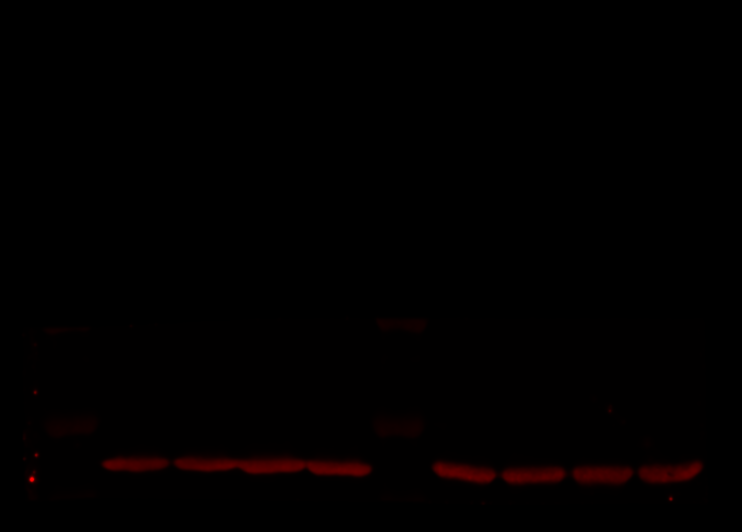


**Supplementary data figure 6a**

GFP probe (five replicates displayed):


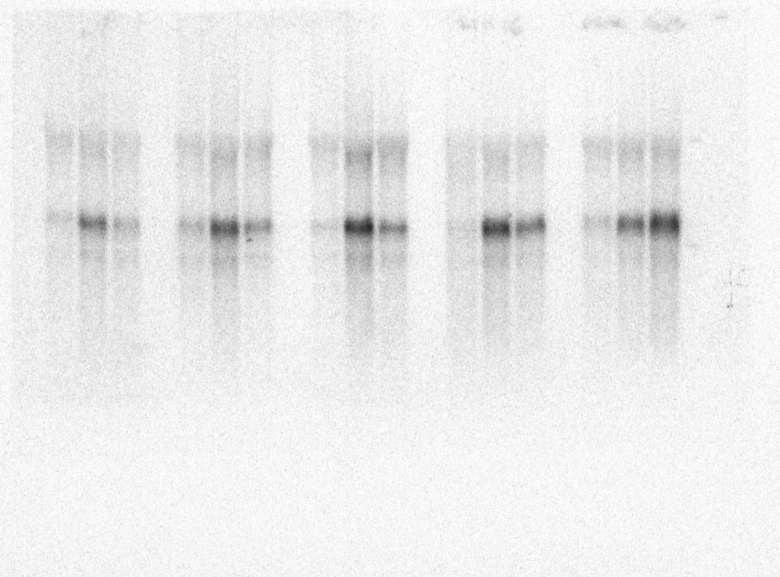


Replicate displayed in figure highlight with black box:


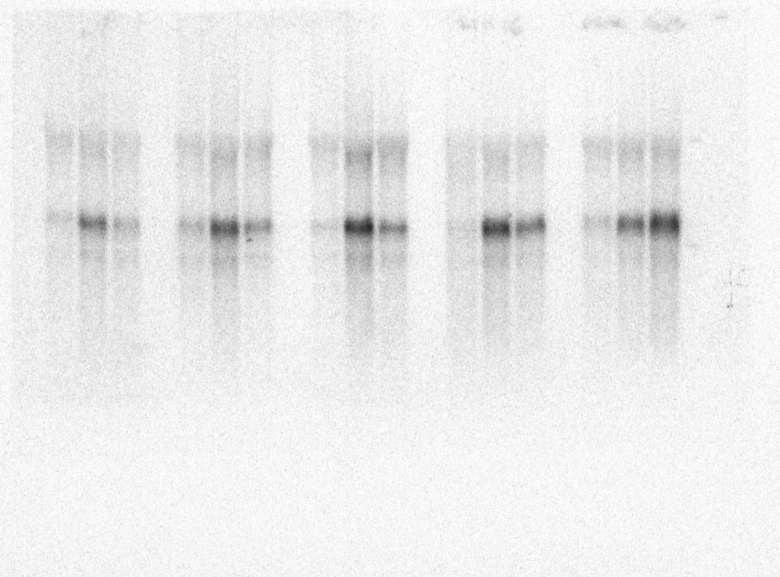


GAPDH probe:


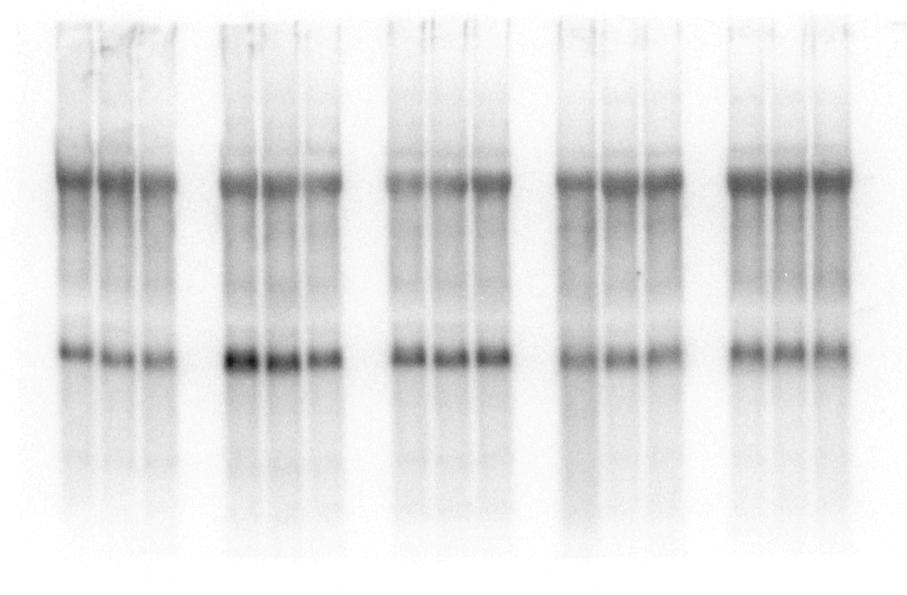

Supplement: Supplementary file 1 — Supplementary Information [file 41467_2022_35268_MOESM1_ESM.docx]
