# Supplementary material for: The PNUTS-PP1 complex acts as an intrinsic barrier to herpesvirus KSHV gene expression and replication
Source: Nat Commun. 2022 Dec 2;13:7447. doi: 10.1038/s41467-022-35268-4 (PMC9718767; doi:10.1038/s41467-022-35268-4)
Supplement: Supplementary file 3 — Reporting Summary [file 41467_2022_35268_MOESM3_ESM.pdf]

nature portfolio

Corresponding author(s): Nicholas Conrad

Last updated by author(s): 11/21/2022

Reporting Summary

Nature Portfolio wishes to improve the reproducibility of the work that we publish. This form provides structure for consistency and transparency in reporting. For further information on Nature Portfolio policies, see our Editorial Policies and the Editorial Policy Checklist.

Statistics

For all statistical analyses, confirm that the following items are present in the figure legend, table legend, main text, or Methods section.

n/a	Confirmed	
☐	☒	The exact sample size (*n*) for each experimental group/condition, given as a discrete number and unit of measurement
☐	☒	A statement on whether measurements were taken from distinct samples or whether the same sample was measured repeatedly
☐	☒	The statistical test(s) used AND whether they are one- or two-sided<br>*Only common tests should be described solely by name; describe more complex techniques in the Methods section.*
☒	☐	A description of all covariates tested
☐	☒	A description of any assumptions or corrections, such as tests of normality and adjustment for multiple comparisons
☐	☒	A full description of the statistical parameters including central tendency (e.g. means) or other basic estimates (e.g. regression coefficient) AND variation (e.g. standard deviation) or associated estimates of uncertainty (e.g. confidence intervals)
☐	☒	For null hypothesis testing, the test statistic (e.g. *F*, *t*, *r*) with confidence intervals, effect sizes, degrees of freedom and *P* value noted<br>*Give P values as exact values whenever suitable.*
☒	☐	For Bayesian analysis, information on the choice of priors and Markov chain Monte Carlo settings
☒	☐	For hierarchical and complex designs, identification of the appropriate level for tests and full reporting of outcomes
☒	☐	Estimates of effect sizes (e.g. Cohen's *d*, Pearson's *r*), indicating how they were calculated

Our web collection on statistics for biologists contains articles on many of the points above.

Software and code

Policy information about availability of computer code

Data collection	Flow cytometry: CellCapture (v3.l)<br>Northern blot images: ImageQuant (v5.2)<br>CRISPR screen Sequencing: Illumina NextSeq 500<br>RNA-Seq, ChIP-Seq library sequencing: Illumina HiSeq 2500<br>qPCR: QuantStudio (vl.5.2)<br>Western blot images: Image Studio (v5.2)
Data analysis	Flowjo v9.9.6<br>Prism (GraphPad) v9<br>IGV (2.9.2)<br>ImageQuant v5.2<br>MAGeCK (VISPR)<br>HISAT (v2.0.l)<br>Sambaba (v0.6.6)<br>featureCounts (vl.4.6)<br>DESeq2 (vl.6.3)<br>Bowtie 2/2.3.3<br>FastQC/0.11.9: data quality check<br>trimgalore/0.6.4: adaptar trimming

Bowtie2/2.3.3: reads alignment
Custom perl script
SAMtools/1.6: File format Conversion
Picard/1.127: PCR duplicates removal
Custom perl script
deeptools suit2.5.0.1: (includes bamCoverage, bamCompare, computeMatrix, and plotProfile), ChIP-Seq and eCLIP analysis

For manuscripts utilizing custom algorithms or software that are central to the research but not yet described in published literature, software must be made available to editors and reviewers. We strongly encourage code deposition in a community repository (e.g. GitHub). See the Nature Portfolio guidelines for submitting code & software for further information.

Data

Policy information about availability of data

All manuscripts must include a data availability statement. This statement should provide the following information, where applicable:

- Accession codes, unique identifiers, or web links for publicly available datasets
- A description of any restrictions on data availability
- For clinical datasets or third party data, please ensure that the statement adheres to our policy

Data generated in support of this study are provided in data figures and supplemental files. CRISPR screen sequencing datasets, eCLIP, RNA-Seq, and ChIP-Seq datasets are available in the GEO super-series GSE201046 [https://www.ncbi.nlm.nih.gov/geo/query/acc.cgi?acc=GSE201046].
Reference genomes used are publicly available at NCBI with the following accession numbers: hg19 [https://www.ncbi.nlm.nih.gov/assembly/GCF_000001405.13/], GRCh38 [https://www.ncbi.nlm.nih.gov/assembly/GCF_000001405.26/], KSHV GQ994935.1 [https://www.ncbi.nlm.nih.gov/nuccore/GQ994935.1].
Source Data are provided with this paper as Source Data.

Human research participants

Policy information about studies involving human research participants and Sex and Gender in Research.

Reporting on sex and gender	N/A
Population characteristics	N/A
Recruitment	N/A
Ethics oversight	N/A

Note that full information on the approval of the study protocol must also be provided in the manuscript.

Field-specific reporting

Please select the one below that is the best fit for your research. If you are not sure, read the appropriate sections before making your selection.

[x] Life sciences   [ ] Behavioural & social sciences   [ ] Ecological, evolutionary & environmental sciences

For a reference copy of the document with all sections, see nature.com/documents/nr-reporting-summary-flat.pdf

Life sciences study design

All studies must disclose on these points even when the disclosure is negative.

Sample size	Sample sizes were chosen to adjust for natural variation in experiment type based on observed magnitude and variation within given samples.
Data exclusions	No data were excluded from the analysis.
Replication	To ensure replication of experimental results, we performed multiple replicates of each experiment and included parallel technical approaches for key assays. For example, the panels in figure 2 demonstrate an increase in KSHV gene expression in two independent cell lines and tested by RNA-Sequencing, RT-qPCR, and northern blotting analysis.
Randomization	Identical cell populations were randomly assigned to control or treatment for individual experiments.
Blinding	No blinding was done.

Reporting for specific materials, systems and methods

We require information from authors about some types of materials, experimental systems and methods used in many studies. Here, indicate whether each material, system or method listed is relevant to your study. If you are not sure if a list item applies to your research, read the appropriate section before selecting a response.

Materials & experimental systems

n/a	Involved in the study
☐	☒ Antibodies
☐	☒ Eukaryotic cell lines
☒	☐ Palaeontology and archaeology
☒	☐ Animals and other organisms
☒	☐ Clinical data
☒	☐ Dual use research of concern

Methods

n/a	Involved in the study
☐	☒ ChIP-seq
☐	☒ Flow cytometry
☒	☐ MRI-based neuroimaging

Antibodies

Antibodies used	Western blotting:<br>Rabbit polyclonal anti-PNUTS Bethyl Laboratories Cat#: A300-439A-M<br>Mouse monoclonal anti-PNUTS BD Biosciences Cat#: 611060<br>Rabbit polyclonal anti-CPSF73 Bethyl Laboratories Cat#: A301-091A<br>Mouse monoclonal anti-beta actin Abeam Cat#ab6276; RRID:AB_2223210<br>Rabbit polyclonal anti-Flag Sigma Cat#: F7425; RRID:AB_ 439687<br>Goat anti-mouse IRDye Cat#:926-68020; RRID:AB_10706161<br>Goat anti-mouse IRDye Cat#:926-32210; RRID:AB_621842<br>Goat anti-rabbit IRDye Cat#: 926-32211; RRID:AB_621843<br>Goat anti-rabbit IRDye Cat#: 926-68021; RRID:AB_10706309<br>eCLIP:<br>Rabbit polyclonal anti-PNUTS Bethyl Laboratories Cat#: A300-439A-M<br>ChIP:<br>Normal Rabbit lgG polyclonal Millipore Cat#: 12-370<br>Rabbit polyclonal anti-RPB3 Millipore Cat#: ABE999
Validation	No primary antibodies were used in this study; all antibodies are commercially available and validated by the manufacturers.<br>Rabbit polyclonal anti-PNUTS Bethyl Laboratories Cat#: A300-439A-M - IHC, Western blot, and IP commercial validations. Validated in-house by western blotting.<br>Mouse monoclonal anti-PNUTS BD Biosciences Cat#: 611060 (RRID: AB_398373): Western blot, immunofluorescence testing by manufacturer. Tested in-house by western blotting.<br>Rabbit polyclonal anti-CPSF73 Bethyl Laboratories Cat#: A301-091A, commercially validated for IP and Western blot.<br>Mouse monoclonal anti-beta actin Abeam Cat#ab6276; RRID:AB_2223210, validated by manufacturer by knockout testing, for ICC/IF and western blot.<br>Rabbit polyclonal anti-Flag Sigma Cat#: F7425; RRID:AB_ 439687, validated by manufacturer for use in dot blots, IPs, indirect immunofluorescence, and western blot.<br>Goat anti-mouse IRDye Cat#:926-68020; RRID:AB_10706161, validated by manufacturer for use as western blot secondary antibody.<br>Goat anti-mouse IRDye Cat#:926-32210; RRID:AB_621842, validated by manufacturer for use as western blot secondary antibody.<br>Goat anti-rabbit IRDye Cat#: 926-32211; RRID:AB_621843 validated by manufacturer for use as western blot secondary antibody<br>Goat anti-rabbit IRDye Cat#: 926-68021; RRID:AB_10706309 validated by manufacturer for use as western blot secondary antibody<br><br>Normal Rabbit lgG polyclonal Millipore Cat#: 12-370, validated by manufacturer for use in IP and western blotting.<br>Rabbit polyclonal anti-RPB3 Millipore Cat#: ABE999, validated by manufacturer for use in western blotting, previously published for use in ChIP assays (e.g. DOI:https://doi.org/10.1016/j.molcel.2020.04.024)

Eukaryotic cell lines

Policy information about cell lines and Sex and Gender in Research

Cell line source(s)	HEK293A-TOA were developed as described in Sahin et al., 2010.<br>293T and HCT116 cells were sourced from ATCC.<br>iSLK cells were from Dr. Rolf Renne, constructed as decribed in Brulois et al., 2012 and Myoung and Ganem 2011.<br>TREx-BCBLl and TREx-RTA-BCBLl were both from Dr. Jae Jung and described in Nakamura et al., 2003.
Authentication	Cell lines were used as received and not tested for authentication. For experiments, cells were discarded before reaching high-passage numbers to maintain integrity.

Mycoplasma contamination	All lines were routinely tested for Mycoplasma contamination and discarded if tests were positive or inconclusive.
Commonly misidentified lines (See ICLAC register)	No commonly misidentified lines were used

ChIP-seq

Data deposition

☒ Confirm that both raw and final processed data have been deposited in a public database such as GEO.

☒ Confirm that you have deposited or provided access to graph files (e.g. BED files) for the called peaks.

Data access links
May remain private before publication.

https://www.ncbi.nlm.nih.gov/geo/query/acc.cgi?acc=GSE201046

Files in database submission

CRISPR Screen
processed data file raw file
AMD_3rep.sgrna_summary.txt Anne_input_Sl_Rl_00l .fastq .gz
AMD_3rep.sgrna_summary.txt Anne_sort_S2_R1_001.fastq.gz
AMD_3rep.sgrna_summary.txt AMD_Input2_Sl_R1_001.fastq.gz
AMD_3rep.sgrna_summary.txt AMD_sort2_S2_R1_001.fastq .gz
AMD_3rep.sgrna_summary.txt AMD_input3_S3_R1_001.fastq.gz
AMD_3rep.sgrna_summary.txt AMD_sort3_S4_R1_001.fastq.gz

RNA-Seq
processed data file raw file
tONT tOP .deseq2.txt X_l_Sl_Rl_00l.fastq.gz
tONT tOP .deseq2.txt X_2_S2_Rl_001.fastq.gz
tONT tOP .deseq2.txt X_3_S3_Rl_001.fastq.gz
tONT_tOP.deseq2.txt X_ 4_S4_Rl_001.fastq.gz
tONT tOP .deseq2.txt X_S_SS_Rl_00l.fastq.gz
tONT tOP .deseq2.txt X_6_S6_Rl_001.fastq.gz
t8NT_t8P.deseq2.txt X_7 _S7 Rl_00l.fastq.gz
t8NT _t8P .deseq2.txt X_8_S8_Rl_001.fastq.gz
t8NT _t8P .deseq2.txt X_9_S9 _Rl_00l.fastq.gz
t8NT _t8P .deseq2.txt X_l0_SlO_Rl_00l.fastq.gz
t8NT _t8P .deseq2.txt X_l l_Sll_Rl_00l.fastq.gz
t8NT _t8P .deseq2.txt X_12_S12_Rl_001.fastq.gz
t24NT _t24P .deseq2.txt S13_Sl_Rl_001.fastq.gz
t24NT _t24P .deseq2.txt S14_S2_Rl_001.fastq.gz
t24NT _t24P .deseq2.txt S15_S3_Rl_001.fastq.gz
t24NT _t24P .deseq2.txt S16_S4_Rl_001.fastq.gz
t24NT_t24P.deseq2.txt Sl 7 SS_Rl_00l.fastq.gz
t24NT t24P .deseq2.txt S18_S6_Rl_001.fastq .gz
t48NT_t48P.deseq2.txt S19_S7 _Rl_00l.fastq.gz
t48NT t48P .deseq2.txt S20_S8_Rl_001.fastq .gz
t48NT t48P .deseq2.txt S21_S9 _Rl_00l.fastq .gz
t48NT t48P .deseq2.txt S22_ Sl0 _Rl_ 001.fastq .gz
t48NT t48P .deseq2.txt S23 _ Sll_Rl_ 001.fastq .gz
t48NT t48P .deseq2.txt S24 _ S12_Rl_ 001.fastq .gz

eCLIP
processed data file raw file
045inp13_S33.CombinedlD.merged.r2.norm.neg.bw 045inp13_S33.fastq .gz
0451P13_S37.CombinedlD.merged.r2 .norm.neg.bw 0451P13_S37.fastq.gz
045inp14_S34.CombinedlD.merged.r2.norm.neg.bw 045inp14_S34.fastq .gz
0451P14_S38.CombinedlD.merged.r2 .norm.neg.bw 0451P14_S38.fastq.gz
PROCESSED DATA FILES
file name file type file checksum
Rep_A_NT.bw bigwig e8cba7c26a34e216b0397f57591f7a47
Rep_B_NT.bw bigwig 8417532ff9af5a91a8e4dc2169053372
Rep_A_Pl.bw bigwig 713c327a7b4b534a5fab556fd706fbd9
Rep_B_Pl.bw bigwig 8c9cc8ca896d2a9b896dad55f29651da
Rep_A_P2.bw bigwig 447f321bbcbdb5079b4b802c18344ae3
Rep_B_P2.bw bigwig 57c97dd77e2413314728af6e8d47b71c
RAW FILES
file name file type
Rep_A_lnput_NT S4_Rl_001.fastq.gz fastq

Rep_A_lnput_Pl_SS_Rl_00l.fastq.gz fastq
Rep_A_lnput_P2_S6_Rl_001.fastq.gz fastq
Rep_A_NT_Sl_Rl_00l.fastq.gz fastq
Rep_A_Pl_S2_Rl_001.fastq.gz fastq
Rep_A_P2_S3_Rl_001.fastq.gz fastq
Rep_B_lnput_NT Sl0_Rl_00l.fastq.gz fastq
Rep_B_lnput_Pl_Sll_Rl_00l.fastq.gz fastq
Rep_B_lnput_P2_S12_Rl_001.fastq.gz fastq
Rep_B_NT_S7 _Rl_00l.fastq.gz fastq
Rep_B_Pl_S8_Rl_001.fastq.gz fastq
Rep_B_P2_S9_Rl_001.fastq.gz fastq

ChIP-Seq in HCT116
PROCESSED DATA FILES
file name
Rep_A_NT.bw
Rep_B_NT.bw
Rep_A_Pl.bw
Rep_B_Pl.bw
Rep_A_P2.bw
Rep_B_P2.bw
RAW FILES
file name
Rep _A_l nput_NT _ S4 Rl_ 001. fastq.gz
Rep_A_lnput_Pl_SS_Rl_00l.fastq.gz
Rep_A_lnput_P2_S6_R1_001.fastq.gz
Rep_A_NT_Sl_Rl_0Ol.fastq.gz
Rep_A_Pl_S2_Rl_001.fastq.gz
Rep_A_P2_S3_R1_001.fastq.gz
Rep_B_lnput_NT SlO_Rl_0Ol.fastq.gz
Rep_B_lnput_Pl_Sll_Rl_0Ol.fastq.gz
Rep_B_lnput_P2_S12_Rl_001.fastq.gz
Rep_B_NT_S7 Rl_00l.fastq.gz
Rep_B_Pl_S8_Rl_001.fastq.gz
Rep_B_P2_59_Rl_001.fastq.gz
ChIP-Seq in iSLK
PROCESSED DATA FILES
file name
Rl_NT _subtract_deeptools.bw
Rl_Pl_subtract_deeptools.bw
Rl_P2_subtract_deeptools.bw
R2_NT subtract_deeptools.bw
R2_Pl_subtract_deeptools.bw
Rep_B_P2.bwR2_P2_subtract_deeptools.bw
RAW FILES
file name
R 1_1 nput_NT _54_Rl_ 001. fastq .gz
Rl_lnput_Pl_S5_R1_001.fastq.gz
R 1_1 nput_P2_56 Rl_ 001. fastq .gz
Rl_NT _Sl_Rl_00l.fastq.gz
Rl_Pl_S2_R1_001.fastq.gz
Rl_P2_53_Rl_001.fastq.gz
R2_1nput_NT Sl0_Rl_00l.fastq.gz
R2_1nput_Pl_Sll_Rl_001.fastq.gz
R2_1nput_p2_512_R1_001.fastq.gz
R2_NT 57 _Rl_00l.fastq.gz
R2_Pl_S8_R1_001.fastq.gz
R2_P2_ 59 _Rl_ 001. fastq.gz

Genome browser session
(e.g. UCSC)

Provide a link to an anonymized genome browser session for "Initial submission" and "Revised version" documents only, to enable peer review. Write "no longer applicable" for "Final submission" documents.

Methodology

Replicates

For both Ch IP-Seq experiments (in the HCT116 reporter cells and in the iSLK cells), two biological replicates were performed, with strong agreement between biological replicates confirmed with principle component plot analysis to validate.

Sequencing depth

ChIP-Seqs in both cell lines were performed in the same manner: single-end sequencing, 75bp reads, with 400 million reads per ChIP shared between 12 samples overall (two biological replicates, 3 IP samples and 3 input samples).
iSLK ChiP-Seq
Sample Name Seqs (TOTAL reads in Millions)
Rl_NT_Sl_Rl_00l 34.7
Rl_Input_NT_S4_Rl_00140.0
R2_NT_S7 _Rl_00l 48.4
Rl_Pl_S2_R1_001 44.0
Rl_Input_Pl_S5_Rl_001 40.2
Rl_P2_53_R1_001 38.3
Rl_Input_P2_56_Rl_00146.3
R2_1nput_NT_S10_Rl_00149.9
R2_Pl_S8_R1_001 39.2
R2_P2_59_R1_001 36.1
R2_1nput_Pl_Sll_Rl_001 50.5
R2_1nput_P2_512_Rl_001 46.9

Sample Name M Seqs (UNIQUELY MAPPED reads in Millions)
Rl_NT_Sl_Rl_00l 28.8
Rl_Input_NT_S4_Rl_001 34.0
R2_NT_S7 _Rl_00l 38.7
Rl_Pl_S2_R1_001 35.8
Rl_Input_Pl_S5_Rl_001 35.1
Rl_P2_53_R1_001 30.0
Rl_Input_P2_56_R1_001 39.7
R2_1nput_NT_S10_Rl_00143.0
R2_Pl_S8_R1_001 32.9
R2_P2_59_R1_001 30.6
R2_1nput_Pl_Sll_Rl_001 43.5
R2_1nput_P2_512_Rl_001 40.3

iSLK ChiP-Seq
Sample Name M Seqs (TOTAL reads in Millions)
Rl_NT_Sl_Rl_00l 34.7
Rl_Input_NT_S4_Rl_00140.0
R2_NT_S7 _Rl_00l 48.4
Rl_Pl_S2_Rl_00144.0
Rl_Input_Pl_S5_R1_001 40.2
Rl_P2_S3_R1_001 38.3
Rl_Input_P2_S6_R1_001 46.3
R2_Input_NT_S10_R1_001 49.9
R2_Pl_S8_R1_001 39.2
R2_P2_S9_R1_001 36.1
R2_Input_Pl_Sll_Rl_001 50.5
R2_Input_P2_S12_R1_001 46.9

Sample Name M Seqs (UNIQUELY MAPPED in Millions)
Rl_NT_Sl_Rl_00l 28.8
Rl_Input_NT_S4_Rl_001 34.0
R2_NT_S7 _Rl_00l 38.7
Rl_Pl_S2_R1_001 35.8
Rl_Input_Pl_S5_R1_001 35.1
Rl_P2_S3_R1_001 30.0
Rl_Input_P2_S6_R1_001 39.7
R2_Input_NT_S10_R1_001 43.0
R2_Pl_S8_R1_001 32.9
R2_P2_S9_R1_001 30.6
R2_Input_Pl_Sll_R1_001 43.5
R2_Input_P2_S12_R1_001 40.3

HCT ChIP Seq
Sample Name M Seqs (TOTAL)
Rl_NT Sl_Rl_00l 42.3
Rl_Input_NT_S4_R1_00147.6
R2_NT S7 _Rl_00l 43.7
Rl_Pl_S2_R1_00142.7
Rl_Input_Pl_S5_R1_00143.5
Rl_P2_S3_R1_001 36.0

	Rl_Input_P2_S6_R1_001 42.3<br>R2_Input_NT_S10_R1_001 39.1<br>R2_Pl_S8_R1_00141.4<br>R2_P2_S9_R1_00144.9<br>R2_Input_Pl_Sll_R1_001 38.8<br>R2_Input_P2_S12_R1_00141.7<br><br>Sample Name M Seqs (UNIQUELY MAPPED)<br>Rl_NT Sl_Rl_00l 36.2<br>Rl_Input_NT_S4_R1_001 39.6<br>R2_NT_S7_R1_00134.7<br>Rl_Pl_S2_R1_001 36.1<br>Rl_Input_Pl_S5_R1_001 34.0<br>Rl_P2_S3_R1_001 31.7<br>Rl_Input_P2_S6_R1_001 34.6<br>R2_Input_NT_S10_R1_001 30.5<br>R2_Pl_S8_R1_001 35.3<br>R2_p2_S9_R1_00135.2<br>R2_Input_Pl_Sll_R1_001 31.6<br>R2_Input_P2_S12_R1_001 34.8
Antibodies	Affinity purified rabbit polyclonal antibody against the RPB3 subunit of RNA Polymerase II from Millipore (Catalogue number ABE999).
Peak calling parameters	macs2 callpeak -t {Sample}.filter.sort_nodup.bam -c {Input}.filter.sort_nodup.bam -g hs -f BAM -q le-2 -B --broad -n {prefix}<br>Building index file and mapping parameters:<br>bowtie2-build GRCh38.reporter dm6.fa GRCh38.reporter dm6<br>bowtie2 --mm --threads ${THREADS}-x ${REPORTERIDX} ${WORKDIR}${Sample}_trimmed.fq.gz -S ${ALIGNDIR}${Sample}.sam
Data quality	Data quality :FastQC/0.11.9<br>Adaptar trimming :trimgalore/0.6.4
Software	FastQC/0.11.9: data quality check<br>trimgalore/0.6.4: adaptar trimming<br>Bowtie2/2.3.3: reads alignment<br>SAMtools/1.6: File format Conversion<br>Picard/1.127: PCR duplicates removal<br>MACS2 (v1.2.1): Peak calling<br>deeptools/2.5.0.1: Metagene profiles

Flow Cytometry

Plots

Confirm that:

- [x] The axis labels state the marker and fluorochrome used (e.g. CD4-FITC).
- [x] The axis scales are clearly visible. Include numbers along axes only for bottom left plot of group (a 'group' is an analysis of identical markers).
- [x] All plots are contour plots with outliers or pseudocolor plots.
- [x] A numerical value for number of cells or percentage (with statistics) is provided.

Methodology

Sample preparation	For cell sorting in CRISPR screen, cells were trypsonized and quenched before spinning down at 800xg at 4C. Pellets were resuspended in 3%FBS PBS and maintained on ice before the sort. For flow cytometry assays, cells were trypsonized as spun as above, then resuspended in 1% formaldehyde PBS to be fixed overnight at 4C. The next day, cells were spun down again and resuspended in 3% FBS PBS for processing on the Stratedigm SlO00 flow cytometer.
Instrument	Cell sorting was performed on FACS Aria Fusion(BD)<br>Flow cytometry was done with a Stratedigm SlO00.
Software	Collection software: CellCapture (v3.l)<br>Analysis software: FlowJo (v9.9.6)
Cell population abundance	For cell sorting, purity of the sorted population was determined by post-sort purity tests with the same settings. Abundance of sorted portion was determined by cut-off gate, set at ~s% highest GFP population.

Gating strategy

Live cells were gated by Forward vs. Side scatter plots, and singlets by Forward scatter Area vs. Forward scatter Height.

☒ Tick this box to confirm that a figure exemplifying the gating strategy is provided in the Supplementary Information.
